# Role of Auriculotherapy in the Treatment of Temporomandibular Disorders with Anxiety in University Students

**DOI:** 10.1155/2015/430143

**Published:** 2015-10-01

**Authors:** Denise Hollanda Iunes, Érika de Cássia Lopes Chaves, Caroline de Castro Moura, Bruna Côrrea, Leonardo César Carvalho, Andreia Maria Silva, Emília Campos de Carvalho

**Affiliations:** ^1^Federal University of Alfenas, Alfenas, MG, Brazil; ^2^University of São Paulo, Ribeirão Preto, SP, Brazil

## Abstract

*Introduction*. The aim of this study was to evaluate the role of auriculotherapy with mustard seeds in the treatment of temporomandibular disorders (TMDs), anxiety, and electromyographic (EMG) activity in university students. *Methodology*. The State Trait Anxiety Inventory (STAI), Research Diagnostic Criteria (RDC) for TMDs (RDC/TMDs), and electromyography were used in this study of 44 college students with high levels of anxiety and TMDs. The subjects were divided into two groups: an auriculotherapy (AA) group (*n* = 31) and an AA sham group (*n* = 13). The mustard seeds were applied to the shenmen, rim, sympathetic, brain stem, and temporomandibular joint (TMJ) points in the AA group and to sham points in the external ear and wrist in the AA sham group. The treatment protocol was 10 sessions (two treatments per week). *Results*. Anxiety (*p* < 0.01) was significantly reduced in the AA group. This group also showed a decrease in tender points in the mandibular posterior region (*p* = 0.04) and in the right side of the submandibular region (*p* = 0.02). Complaints of bilateral pain were reduced in the temporal tendon (*p* ≤ 0.01) and in the left side of the ATM (*p* < 0.01). In addition, electromyographic (EMG) activity was reduced during temporal muscle contraction (*p* = 0.03).  *Conclusion*. Auriculotherapy was effective in the treatment of students with anxiety and TMDs.

## 1. Introduction

Temporomandibular disorders (TMDs) are one of the most common causes of orofacial complaints. They have multiple clinical manifestations, but the most frequent are pain in the region of the temporomandibular joint, pain and fatigue of the craniocervical muscles, especially those involved in mastication, limitation and deviations of mandibular movements, the presence of joint sounds [1], headaches, sensitivity to palpation of the masticatory muscles and temporomandibular joints [2], and tinnitus [3]. Given the variety of symptoms, TMDs have been attributed to multiple etiological factors [4], such as anatomical, functional, and psychosocial changes [4, 5]. There is a lack of consensus on whether there is a relationship between anxiety, depression, and TMDs [4].

Pain relief is the main objective of primary therapeutic treatment of patients with TMDs. Treatment strategies include drugs to control chronic pain, physical therapy, surgery, and arthroscopy [6]. Dental approaches include occlusal intraoral devices and occlusal adjustment [7]. Psychosocial interventions [8] and low-frequency laser therapy have also been applied [9]. According to the literature, complementary and integrative practices are often used, in conjunction with conventional treatment [6, 10].

Auriculotherapy or ear acupuncture is a therapeutic acupuncture technique [11] which is based on the idea that pluripotent cell groups contain information on the whole organism and create regional organization centers, which represent different parts of the body, and that stimulation of a reflection point in the auricle for a sufficient duration can relieve the symptoms of a disease [12]. Treatment with auriculotherapy is one of the most popular systemic microacupuncture techniques, with extensive applications [13].

Various studies have demonstrated the potential of auricular therapy in the treatment of a variety of conditions, such as its use in the treatment of TMDs [7] and its symptoms [14], especially pain [15]. Another study found that it improved the quality of life of individuals treated with traditional Chinese medicine (TCM), combined with conventional therapy [6].

Thus, the aim of this study was to evaluate the role of auricular acupuncture in the treatment of TMDs and anxiety in university students and the impact of the treatment on the electromyographic activity of various muscles.

## 2. Methodology

This controlled clinical, randomized, double-blind study was conducted with federal university students attending various health care courses (nursing, physiotherapy, pharmacy, and dentistry). A sociodemographic and clinical questionnaire was used to screen the students for major signs and symptoms of TMDs, such as headache, clicks, masticatory muscle pain, and TMJ pain. Ninety-seven students who reported signs and symptoms of TMDs were selected for the study.

The research was conducted over a 7-month period (October 2013 to May 2014). The inclusion criteria for this study were age being 18 years or over, availability for auriculotherapy sessions, and high levels of anxiety according to the State Trait Anxiety Inventory (STAI) [16]. The exclusion criteria were ear piercings (except a regular earring), inflammation, infection, or injury to the ear, receiving drug treatment for TMDs and anxiety, orofacial pain, or pregnancy.

**Figure 1:**
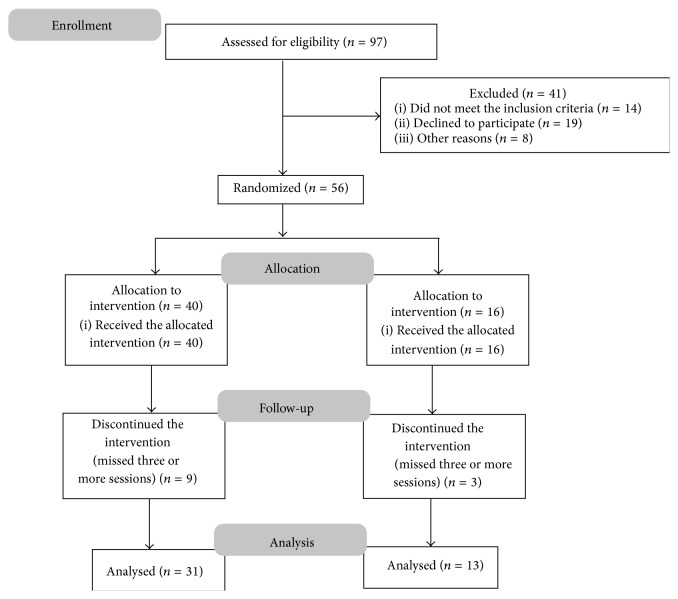
Flowchart of the participants.

Recruitment and enrollment of the participants is described in a trial flow diagram (Figure 1). Fifty-six volunteers fulfilled the eligibility criteria and were divided into two groups by simple randomization: an auriculotherapy group (AA) (*n* = 40) and a sham AA group (*n* = 16). During the intervention, some subjects dropped out. The postintervention reevaluation included 31 individuals in the AA group and 13 subjects in the sham AA group.

Fifty-six volunteers fulfilled the eligibility criteria and were evaluated at baseline and received a number. Then by a simple selection in a draw fewer volunteers were separated for the sham AA group (*n* = 16) and getting the other for the auriculotherapy group (AA) (*n* = 40).

The study subjects were evaluated before the first auriculotherapy session and after the 10th session by the same trained examiner who had no knowledge of the type of treatment applied. The instruments used in this evaluation were the STAI [16], I axis of the Research Diagnostic Criteria for TMDs (RDC/TMDs) [17], and surface electromyography (sEMG) measurements of the electrical activity of the bilateral trapezius, masseter, and temporal muscles.

The STAI was translated and adapted for the Portuguese language [18]. The STAI is composed of two parts, with 20 items for assessing trait anxiety and 20 for assessing state anxiety. The answers are scored on a Likert 4-point scale. The score ranges from 20 to 80 points, with 0–30 indicating a low level of anxiety, 31–49 denoting a medium level of anxiety, and 50 or more indicating a high level of anxiety [16].

The RDC/TMDs allowed standardized assessment [17]. This instrument is divided into two axes. Axis I is the physical examination for the classification of subtypes of TMDs into three groups: muscle disorders (group Ia and Ib myofascial pain and myofascial pain with limited opening), disk displacement dysfunction (group II), and joint disorders (IIIa, arthralgia; IIIb, TMJ; and IIIc, osteoarthritis). The reliability of the RDC/TMDs was previously tested [19, 20], and the instrument was translated and validated officially for the Portuguese language [21]. To measure the intensity of pain in the evaluated points, we used a visual analog scale (VAS), where 0 was no pain and 10 denoted severe pain [7].

The EMG signals of the masseter and anterior temporal muscles were collected by disposable bipolar surface electrodes (Hal and Hal, São Paulo, Brazil). The EMG signals of the trapezius and reference muscles were collected using Meditrace monopolar electrodes with an AgCl catchment surface and a diameter of 10 mm (Tyco/Kendall, Mansfield, Canada). The monopolar electrodes were positioned parallel to each other at a distance of 20 mm center to center, along the fibers of the muscles described above, as prescribed earlier [22].

All the electromyography signals were captured with the EMG-Brazil Model 800C. In this model, six channels are configured to receive the EMG signals with a digital band-pass filter, a cutoff frequency of 20–500 Hz, and final gain of 1000 times. Another channel is configured to receive signals from the load cell used for maximum voluntary isometric contraction. All the channels have a sampling frequency of 2000 Hz. The system features specific software for signal acquisition and storage in data files. The EMG signals of the trapezius muscle were collected at rest, during isometric contraction (bilateral and unilateral) against gravity, and during maximum voluntary isometric contraction [23] using a load cell of 200 kgf. For the masseter and temporal muscles, the EMG signals were collected in the mandibular rest position and during maximal voluntary isometric contraction [24]. All the data were collected in triplicate while the subjects contracted their muscles for 10 sec, at intervals of 60 sec. In the analysis, we used the data collected during 2–7 sec. During the collection of the EMG data, the volunteers sat on a chair, with their feet flat on the floor. They rested their hands on their legs, with their shoulders relaxed and their head parallel to the Frankfurt line. They were directed to look straight ahead.

The EMG signals were processed with a specific algorithm, using programmed routines in MatLab software (Algorithm 1). Quantification of the signal was performed by RMS amplitude, as recommended to evaluate the level of muscle activity [25].

We established a protocol to determine the application of the points in the auriculotherapy. The protocol was based on personal clinical experience, the Standards for Reporting Interventions in Clinical Trials of Acupuncture [26], and the literature [27, 28]. The protocol was later submitted for refining to four judges with 2–10 years of accreditation and experience in auriculotherapy. Interventionists had training in auriculotherapy and at least two years of experience in the area.

**Figure 2:**
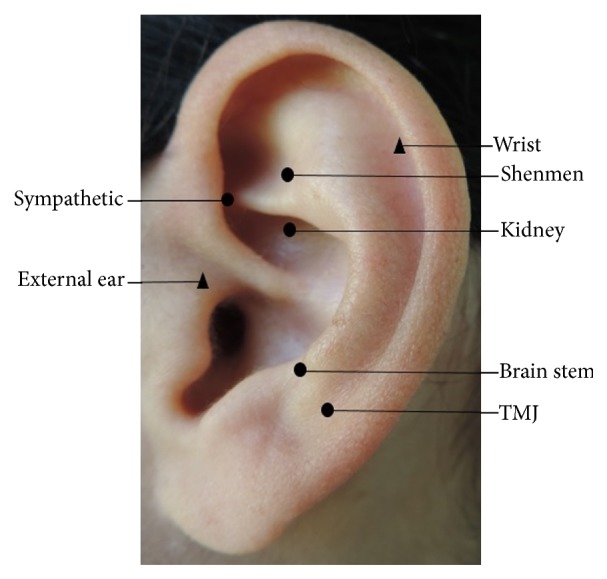
Auricular points used in the intervention: AA group (circle) and sham AA group (triangle). These points were used in the right and left ears alternately.

The auriculotherapy used mustard seeds, which were attached to the skin with Micropore tape. Each volunteer underwent 10 sessions, twice a week (Monday and Thursday) for 6 weeks, with an alternate ear used each application. Prior to the placement of the mustard seeds, the subject's ear was cleaned with 70% ethyl alcohol. During the placement of the seeds, the volunteer remained sitting on a chair with a back support. As a constant pressure stimulus on the point is needed for the intervention to have the expected effect, the volunteer was instructed to press each auricular point at least 5 times a day, applying pressure for 1 min to every point [29] or until the pressure produced localized pain or discomfort [30]. The AA group received five points per subject per session being the shenmen, kidney, sympathetic, brain stem [27, 28], and TMJ [27]. These points have sedative and tranquilizer effects [11, 27, 31]. The sham AA group received two points per subject per session being the wrist and external ear [27, 28] (Figure 2). These points were chosen because they were far from the group of points the AA group.

The Statistical Package for the Social Sciences (SPSS), version 23.0, was used for the statistical analysis. The Shapiro-Wilk normality test was performed, followed by a* t*-test for data with a normal distribution and a Wilcoxon and Mann-Whitney test for data with a nonnormal distribution. The significance level was 5%. The effect size and the power effect of the sample were calculated with GPower 3.1.7 software (Franz Faut, Universität Kiel Germany, 2008). A small effect size (*d*) was considered 0.20 ≤ *d* < 0.50, a medium effect size was considered 0.50 ≤ *d* < 0.80, and a large effect size was considered *d* ≥ 0.80. In the power analysis, more than 0.80 was needed to denote adequate power [32].

This study was approved by the Research Ethics Committee of the Federal University of Alfenas (Protocol number: 164 590), and it was registered with the Brazilian Registry of Clinical Trials Platform (Protocol number: U111-1147-3083). The volunteers received information regarding their participation in this research study and they signed free and informed consent documents.

## 3. Results

Forty-four college students participated in this study: 31 were assigned to the AA group and 13 were in the sham AA group. In the AA group, 93.55% (*n* = 28) were women, and 9.67% (*n* = 3) were men. The mean ± SD age of the study participants was 21.61 ± 3.27 years. In the sham AA, 100% (*n* = 13) of the subjects were women, and the mean ± SD age of the study participants was 20.87 ± 1.50 years. According to the RDC/TMDs, all the participants were classified as category Ia. In the AA group and sham AA group, 33% and 15.38% of subjects, respectively, were also classified as having a category IIIa TMD. In the AA group, the anxiety state of the subjects was significantly reduced after the application of auriculotherapy (Table 1).

The results of the evaluation of the mobility of mouth movements using the RDC/TMD are shown in Table 2. No statistically significant changes were identified, even in the maximal passive opening movement in the sham AA group. Although the values of maximum passive mouth opening were significant, it exhibited a low power and a moderate effect size. In the initial assessment of both groups, none of the subjects had limitations in the evaluated movements.

The RDC/TMDs were used to evaluate the tender points of the masticatory muscles and TMJs and palpation of the trapezius muscle. The results are shown in Table 3. In the AA group, pain was significantly reduced in the five points assessed, together with a high power and medium to high effect size. The other points showed clinical improvement. In the sham AA group, 46% of those evaluated reported increased pain at the evaluated points, with statistical significance.

Table 4 shows the comparison of the intragroup and intergroup analyses of the EMG activity in the AA and sham AA groups. Only the bilateral contraction of the temporalis muscle in the AA group showed a significant difference between the pre- and postintervention, with a low power effect. However, in the posttreatment intergroup analysis, the EMG activity of the trapezius and temporalis muscles at rest and during contraction showed statistical significance.

## 4. Discussion

In TCM, disease is seen as an imbalance in the meridians. Therefore, the processing based on this analysis has the potential to provide a strategy for overall system management for TMDs [6], mainly with a view to reduce pain [33] and anxiety [11]. As noted previously, there is a need for different studies to find common auricular acupuncture points to create an international standard of clinical research that facilitates replication and dissemination of the results [13]. The establishment of the protocol for the treatment of TMDs in this study can aid this goal, although further multicenter, longitudinal studies with larger samples are needed in this area.

In the present study, auriculotherapy significantly reduced anxiety and provided pain relief. It also reduced the electrical activity of the trapezius and temporal muscles. Anxiety can contribute to the development of a TMD or be the result of this disorder and its perpetuation [34]. In this study, all the volunteers with high levels of anxiety had TMDs. Other researchers also observed a reduction in anxiety after auriculotherapy [11, 35, 36], pointing to the potential of this technique in the control of various clinical conditions.

Anxiety is an increasingly common disorder in people's lives today [37]. It is treated with anxiolytic psychotropic drugs, often at the request of the patient leading to unnecessary and inappropriate treatment [38]. The use of integrative/complementary treatments, including auriculotherapy, has been investigated to reduce the dependence on such drugs. The former can be integrated with traditional therapy or even replace it, as integrative/complementary treatments do not have side effects. They are also inexpensive and easy to administer.

TMD-related pain can be chronic, with the dysfunction affecting not only peripheral nervous system, but also the central nervous system, thereby producing a widespread perception of pain [39]. This type of pain is often difficult to treat and is accompanied by emotional components, such as anxiety and/or depression. The symptoms of anxiety are strongly related to muscle pain [40]. Joint symptoms were also observed in the present study.

The goal of all TMD treatment is to minimize pain. In the present study, the therapy was effective in reducing pain, with statistical significance for five of the points assessed. The findings corroborate those of an earlier study [7], which demonstrated the contribution of Eastern therapies in decreasing TMD-related pain. Treatments for TMDs must act in a holistic manner, both on the physical and emotional symptoms. In the current study, anxiety and pain were significantly reduced in the subjects who received auriculotherapy. In addition, the electrical activity of the muscles was significantly reduced in the intergroup analysis.

The anxiety and pain associated with TMDs can trigger hyperactivity and altered muscle mechanics, which can perpetuate the muscle pain [41]. Therefore, a muscle evaluation is important to establish the diagnosis and treatment of TMDs. In many cases, TMDs may cause inflammation of the joints, followed by biomechanical changes, which give rise to pain in the affected region. A previous study found that TMDs may be related to abnormal processing of pain in the trigeminal system [41]. Other studies pointed to the relationship between mastigatory and cervical muscle tenderness associated emotional changes [42, 43]. They found that this resulted in emotional changes, which predisposed individuals to orofacial pain and TMDs [42, 43]. In the current study, the efficacy of the treatment was assessed by evaluating the presence of pain in both groups and the EMG activity. The treatment was effective, as shown by the improvement in tender points and the electrical activity of the trapezius muscle over time in the intergroup analysis. Therefore, it is believed that the auriculotherapy may have served as a mechanism of muscle modulatory activity, as reported previously [34].

A previous study reported that anxiety and stress contributed to the development of TMDs and that it increased the recruitment of the anterior temporal muscle, sternocleidomastoid, and upper trapezius, in addition to the excitability of the trigeminal-neck reflex, causing pain and trigger points in the muscles, leading to a vicious cycle [44]. In the present study, after the intervention, the EMG activity of the descending trapezius and anterior temporal decreased during maximal muscle contraction. The reduction in the EMG activity of the temporal muscle was significant in the AA group but not in the sham AA group. No changes in EMG activity were observed in the masseter muscle. A previous study reported that the EMG activity of the descending fibers of the trapezius did not change in individuals without a history of masticatory system dysfunction during maximal effort centric occlusion of the jaw [23]. The lack of EMG activity reported in the previous study explains why anxiety and stress can lead to the development of a vicious cycle that contributes to hyperactivity of the masticatory muscles in adolescents with severe TMD symptoms [24].

The between-group comparison revealed a reduction in EMG activity at rest and during muscle contraction. This reduction in EMG activity may be related to the effect of the auriculotherapy that was observed after the intervention, as auriculotherapy stimulates the peripheral nervous system by promoting local and systemic reflex responses [45, 46]. These responses are mediated by the release of endorphins, serotonin, and noradrenaline released from the endocrine system, immune system, and higher centers in the central control of pain [45, 46].

*Study Limitation*. The limitations of this study were the small sample size, absence of follow-up, and absence of a control group.

## 5. Conclusion

Auriculotherapy helped to reduce anxiety and tender points in the posterior region right mandibular and submandibular, tendon bilateral temporalis, and left TMJ. It also reduced EMG activity during temporal muscle contraction.

## Figures and Tables

**Algorithm 1:**
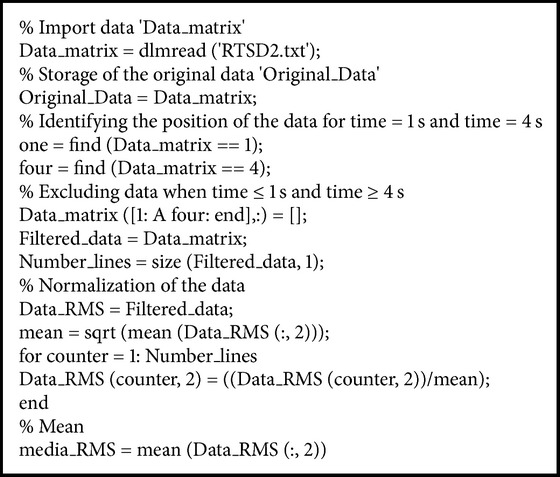
Algorithm used for normalization of the electromyography data.

**Table 1:** Pretreatment and posttreatment comparison of the mean anxiety profile according to the STAI-E in the AA group and sham AA group.

Groups	Pretreatment	Posttreatment	*p* ^*∗*^	*d*
	95% CI			
AA	53.26	45.60	<0.01^*∗*^	0.84^*∗∗*^
(*n* = 31)	48.90–57.62	40.08–51.11		

Sham AA	48.20	47.00	0.58^*∗*^	0.11
(*n* = 13)	43.71–52.68	40.45–53.54		

*d*: effect size; ^*∗*^paired *t* test; ^*∗∗*^power > 80%.

**Table 2:** Mobility evaluation of the mouth movements of the AA and sham AA groups pretreatment and posttreatment.

Mouth movements	AA (*n* = 31)				Sham AA (*n* = 13)			
	Pretreatment	Posttreatment	*p* ^*∗*^	*d*	Pretreatment	Posttreatment	*p* ^*∗*^	*d*
	(95% CI)				(95% CI)			
Passive opening	36.50 32.88–40.21	38.30 35.44–41.31	0.40	0.20	37.30 30.60–44.16	38.40 33.73–43.03	0.96	0.11

Maximum passive opening	50.90 47.59–54.34	51.00 48.73–54.02	0.80	0.01	49.00 45.01–53.13	52.50 48.29–56.62	0.04^*∗*^	0.51

Maximum active opening	47.60 44.51–50.72	47.00 44.28–50.12	0.90	0.08	46.30 41.45–51.16	49.50 46.86–52.05	0.07	0.46

Overlap	3.50 2.81–4.21	3.40 2.51–4.37	0.60	0.05	5.40 4.11–6.80	4.20 2.51–5.94	0.19	0.47

Right lateral deviation	8.40 6.98–9.84	8.20 7.19–9.63	0.70	0.06	7.50 5.48–9.59	9.20 8.01–10.44	0.12	0.57

Left lateral deviation	8.70 7.71–9.73	7.86 6.99–8.80	0.10	0.34	7.40 5.41–9.50	8.50 7.34–9.58	0.30	0.37

Protrusion	5.40 4.37–6.59	4.80 3.76–5.95	0.20	0.21	6.40 5.31–7.60	6.20 5.44–7.01	0.70	0.12

*d*: effect size; ^*∗*^Wilcoxon test.

**Table 3:** Pretreatment and posttreatment comparison of the tender points of the AA and sham AA groups based on the RDC/TMDs and the electromyographic activity of the trapezius muscle.

Points	AA (*n* = 31)				Sham AA (*n* = 13)				Intergroup	
	Pretreatment	Posttreatment	*p*	*d*	Pretreatment	Posttreatment	*p*	*d*	Before (*p*)	After (*p*)
	(95% CI)				(95% CI)					
Right temporal										
A	1.20 0.47–1.93	0.93 0.24–1.62	0.45	0.15	1.34 0.04–2.72	1.77 0.98–2.55	0.51	0.22	0.64	0.01
M	1.00 0.20–1.79	0.86 0.17–1.54	0.57	0.67	0.85 −0.07–1.76	1.08 0.44–1.70	0.76	0.17	0.79	0.16
P	0.96 0.28–1.64	0.86 0.17–1.54	0.66	0.06	0.85 −0.07–1.76	0.77 0.15–1.38	0.74	0.06	0.95	0.60
Left temporal										
A	1.00 0.14–1.85	0.76 0.21–1.30	0.51	0.12	2.15 0.65–3.65	1.23 0.61–1.84	0.20	0.41	0.03	0.07
M	0.93 0.19–1.66	0.82 0.27–1.37	0.54	0.06	1.00 0.07–1.92	1.31 0.40–2.21	0.32	0.21	0.40	0.20
P	0.76 0.02–1.49	0.75 0.21–1.30	0.93	0.01	1.15 0.23–2.07	0.92 2.29–1.55	0.53	0.17	0.09	0.34
Right masseter										
O	2.96 1.99–3.93	1.69 0.90–2.47	0.06	0.54	2.38 0.99–3.77	1.85 0.83–2.85	0.32	0.26	0.50	0.48
B	2.58 1.73–3.43	1.62 0.85–2.38	0.10	0.45	3.08 1.48–4.66	2.23 1.15–3.31	0.37	0.37	0.60	0.20
I	1.58 0.68–2.48	1.00 0.44–1.55	0.29	0.23	4.08 2.52–5.62	1.70 0.63–2.75	0.01^*∗*^	**1.05**	<0.01	0.17
Left masseter										
O	3.10 2.01–4.19	1.96 1.16–2.76	0.08	0.44	1.92 0.51–3.32	2.31 1.06–3.55	0.51	0.18	0.22	0.55
B	2.72 1.77–3.67	2.10 1.26–2.93	0.25	0.26	2.31 1.06–3.55	2.08 0.96–3.19	0.68	0.12	0.71	0.95
I	1.58 0.63–2.53	1.76 0.78–2.73	0.82	0.07	3.54 2.10–4.96	2.61 1.52–3.70	0.17	0.43	<0.01	0.07
Posterior mandibular region										
R	5.00 4.00–5.99	3.44 2.54–4.35	0.04^*∗*^	**0.62**	4.84 3.21–6.47	7.08 6.20–7.94	0.03^*∗*^	**0.96**	0.85	0.00
L	4.69	4.34 3.15–5.53	0.33	0.11	3.85 2.15–5.53	6.38 5.40–7.32	0.01^*∗*^	**1.04**	0.37	0.05
Submandibular region										
R	5.07 3.87–6.26	3.07 2.14–3.99	0.02^*∗*^	**0.70**	5.38 3.70–7.06	5.77 4.20–7.33	0.72	0.14	0.90	<0.01
L	5.00 3.91–3.08	4.21 3.32–5.08	0.14	0.30	5.38 3.54–7.22	5.54 3.85–7.22	0.92	0.05	0.66	0.11
Lateral pterygoid										
R	3.65 2.64–4.66	2.45 1.47–3.42	0.09	0.46	2.08 0.25–3.89	1.23 0.09–2.36	0.18	0.32	0.06	0.09
L	2.00 1.01–2.98	2.51 1.51–3.51	0.42	0.20	2.69 0.80–4.57	1.85 0.45–3.23	0.45	0.30	0.55	0.44
Temporal tendon										
R	4.93 3.85–6.01	2.96 2.04–3.88	0.01^*∗*^	**0.74**	5.31 3.86–6.75	4.30 2.79–5.81	0.17	0.41	0.77	0.11
L	4.55 3.46–5.63	2.31 1.34–3.27	<0.01^*∗*^	**0.83**	5.46 4.21–6.71	3.67 2.00–5.53	0.06	0.69	0.38	0.10
Side portion TMJ										
R	3.52 2.53–4.50	3.17 2.10–4.24	0.48	0.13	3.08 1.86–5.97	2.92 3.20–6.33	0.79	0.06	0.79	0.06
L	3.34 0.96–1.86	1.41 0.68–2.21	<0.01^*∗*^	**0.80**	4.46 2.63–6.68	4.61 2.91–6.31	0.83	0.05	<0.01	0.17
Back of the TMJ										
R	1.83 1.05–2.59	1.45 2.29–4.39	0.70	0.19	3.92 1.22–4.93	4.77 1.43–4.41	0.44	0.28	0.26	0.04
L	0.62 0.34–0.89	0.93 0.31–1.54	0.64	0.22	3.00 1.23–4.76	1.77 0.32–3.21	0.07	0.46	<0.01	0.18
Trapezius										
R	5.03 3.85–6.21	4.17 3.09–5.25	0.16	0.29	5.00 3.08–6.91	6.23 4.61–7.85	0.22	0.42	0.88	0.03
L	4.03 2.93–5.30	3.89 2.74–5.04	0.78	0.05	3.23 1.30–5.16	5.77 4.03–7.49	0.02^*∗*^	**0.84**	0.38	0.06

A: anterior; M: middle; P: posterior; O: origin; B: belly; I: insertion; R: right; L: left; *d*: effect size; ^*∗*^Wilcoxon test; intergroup analysis: Mann-Whitney test.

**Table 4:** Results of the intragroup and intergroup analysis of the EMG activity of the AA group and sham AA group.

Muscles	AA (*n* = 31)				Sham AA (*n* = 13)				Intergroup	
	Before	After	*p* ^*∗*^	*d*	Before	After	*p* ^*∗*^	*d*	Before (*p*)	After (*p*)
	(95% CI)				(95% CI)					
Bilateral trapezius rest										
R	3.33	3.34	0.43	0.06	3.21	3.21	1.00	0.00	0.267	**<0.01^#^**
	3.27–3.39	3.28–3.41			3.00–3.42	3.04–3.38				
L	3.64	3.66	0.12	0.08	3.49	3.50	0.46	0.19	0.899	0.03^#^
	3.55–3.73	3.56–3.75			3.19–3.79	3.25–3.75				
Bilateral masseter rest										
R	3.35	3.66	0.23	0.18	3.24	3.23	0.14	0.03	0.750	0.07
	3.29–3.42	2.99–4.33			3.02–3.46	3.06–3.39				
L	3.63	3.65	0.42	0.09	3.49	3.51	0.91	0.05	0.466	0.15
	3.55–3.72	3.56–3.73			3.11–3.53	3.27–3.75				
Bilateral temporalis rest										
R	3.41	3.40	0.23	0.07	3.32	3.29	0.05	0.08	0.841	0.21
	3.35–3.47	3.37–3.43			3.11–3.53	3.14–3.45				
L	3.58	3.55	0.12	0.14	3.44	3.43	0.11	0.02	0.236	0.02^#^
	3.51–3.66	3.47–3.62			3.21–3.67	3.24–3.61				
Bilateral trapezius contraction										
R	3.23	3.23	0.51	0.00	3.12	3.11	0.23	0.03	0.216	**<0.01^#^**
	3.18–3.29	3.18–3.29			2.93–3.32	3.00–3.23				
L	3.55	3.55	0.47	0.00	3.41	3.38	0.06	0.06	0.548	**<0.01^#^**
	3.48–3.62	3.49–3.62			3.14–3.69	3.17–3.59				
Unilateral trapezius contraction										
R	3.26	3.24	0.71	0.10	3.16	3.11	0.26	0.12	0.636	**<0.01^#^**
	3.18–3.33	3.17–3.31			2.91–3.41	2.98–3.24				
L	3.53	3.54	0.56	0.04	3.345	3.39	0.78	0.13	0.063	**<0.01^#^**
	3.45–3.62	3.46–3.62			2.70–5.36	3.21–3.58				
Bilateral masseter contraction										
R	3.23	3.20	0.09	0.25	3.15	3.10	0.09	0.19	0.675	0.23
	3.18–3.27	3.16–3.24			2.99–3.30	2.99–3.21				
L	3.51	3.51	0.29	0.00	3.39	3.39	0.50	0.00	0.455	0.88
	3.43–3.59	3.44–3.58			3.15–3.62	3.19–3.59				
Temporal bilateral contraction										
R	3.30	3.29	0.40	0.07	3.21	3.18	0.17	0.09	0.362	**<0.01^#^**
	3.25–3.36	3.24–3.34			3.01–3.41	3.05–3.31				
L	3.48	3.46	0.03^*∗*^	0.10	3.35	3.34	0.14	0.03	0.209	0.03^#^
	3.41–3.56	3.39–3.52			3.13–3.56	3.17–3.50				

R: right; L: left; *d*: effect size; ^*∗*^Wilcoxon test; ^#^Mann-Whitney test.
